# Multiscale *In Silico* Study of the Mechanism of Activation of the RtcB Ligase by the PTP1B Phosphatase

**DOI:** 10.1021/acs.jcim.3c01600

**Published:** 2024-01-29

**Authors:** Sayyed Jalil Mahdizadeh, Michael Stier, Antonio Carlesso, Aurore Lamy, Melissa Thomas, Leif A. Eriksson

**Affiliations:** † Department of Chemistry and Molecular Biology, University of Gothenburg, 405 30 Gothenburg, Sweden; ‡ Department of Pharmacology, Sahlgrenska Academy, University of Gothenburg, 413 90 Gothenburg, Sweden; § Università della Svizzera italiana (USI), Faculty of Biomedical Sciences, Euler Institute, Via G. Buffi 13, CH-6900 Lugano, Switzerland; ∥ Department of Bioinformatics and Chemical Communication, Research Institute in Semiochemistry and Applied Ethology, Quartier Salignan, 84400 Apt, France

## Abstract

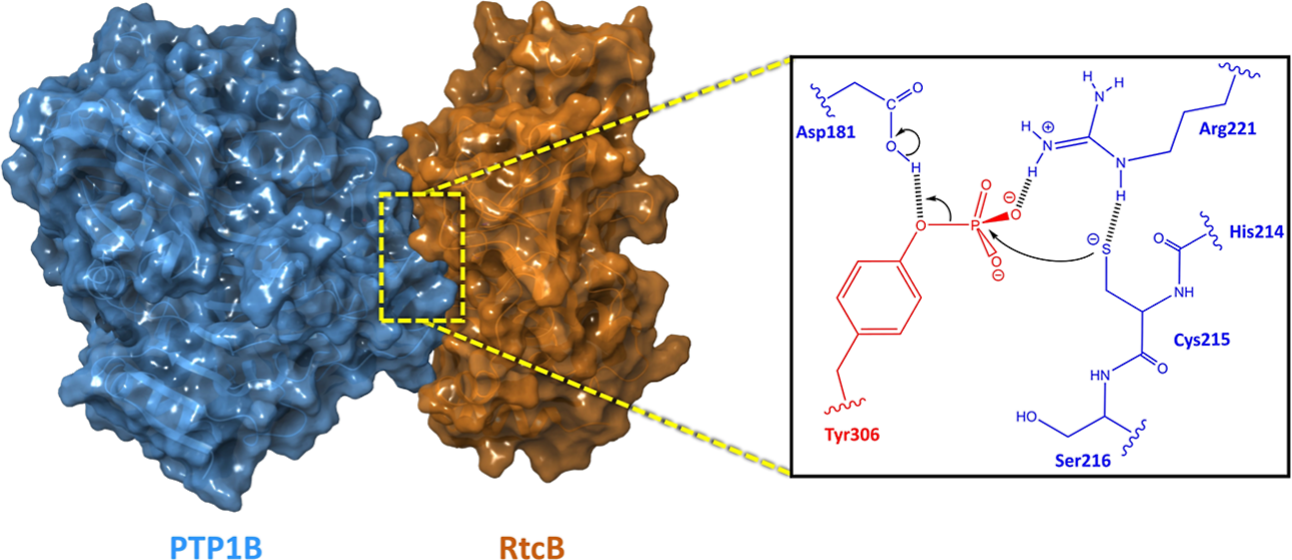

Inositol-requiring enzyme 1 (IRE1) is a transmembrane sensor that is part of a trio of sensors responsible for controlling the unfolded protein response within the endoplasmic reticulum (ER). Upon the accumulation of unfolded or misfolded proteins in the ER, IRE1 becomes activated and initiates the cleavage of a 26-nucleotide intron from human X-box-containing protein 1 (XBP1). The cleavage is mediated by the RtcB ligase enzyme, which splices together two exons, resulting in the formation of the spliced isoform XBP1s. The XBP1s isoform activates the transcription of genes involved in ER-associated degradation to maintain cellular homeostasis. The catalytic activity of RtcB is controlled by the phosphorylation and dephosphorylation of three tyrosine residues (Y306, Y316, and Y475), which are regulated by the ABL1 tyrosine kinase and PTP1B phosphatase, respectively. This study focuses on investigating the mechanism by which the PTP1B phosphatase activates the RtcB ligase using a range of advanced in silico methods. Protein–protein docking identified key interacting residues between RtcB and PTP1B. Notably, the phosphorylated Tyr306 formed hydrogen bonds and salt bridge interactions with the “gatekeeper” residues Arg47 and Lys120 of the inactive PTP1B. Classical molecular dynamics simulation emphasized the crucial role of Asp181 in the activation of PTP1B, driving the conformational change from an open to a closed state of the WPD-loop. Furthermore, QM/MM-MD simulations provided insights into the free energy landscape of the dephosphorylation reaction mechanism of RtcB, which is mediated by the PTP1B phosphatase.

## 1 Introduction

**Figure 1:**
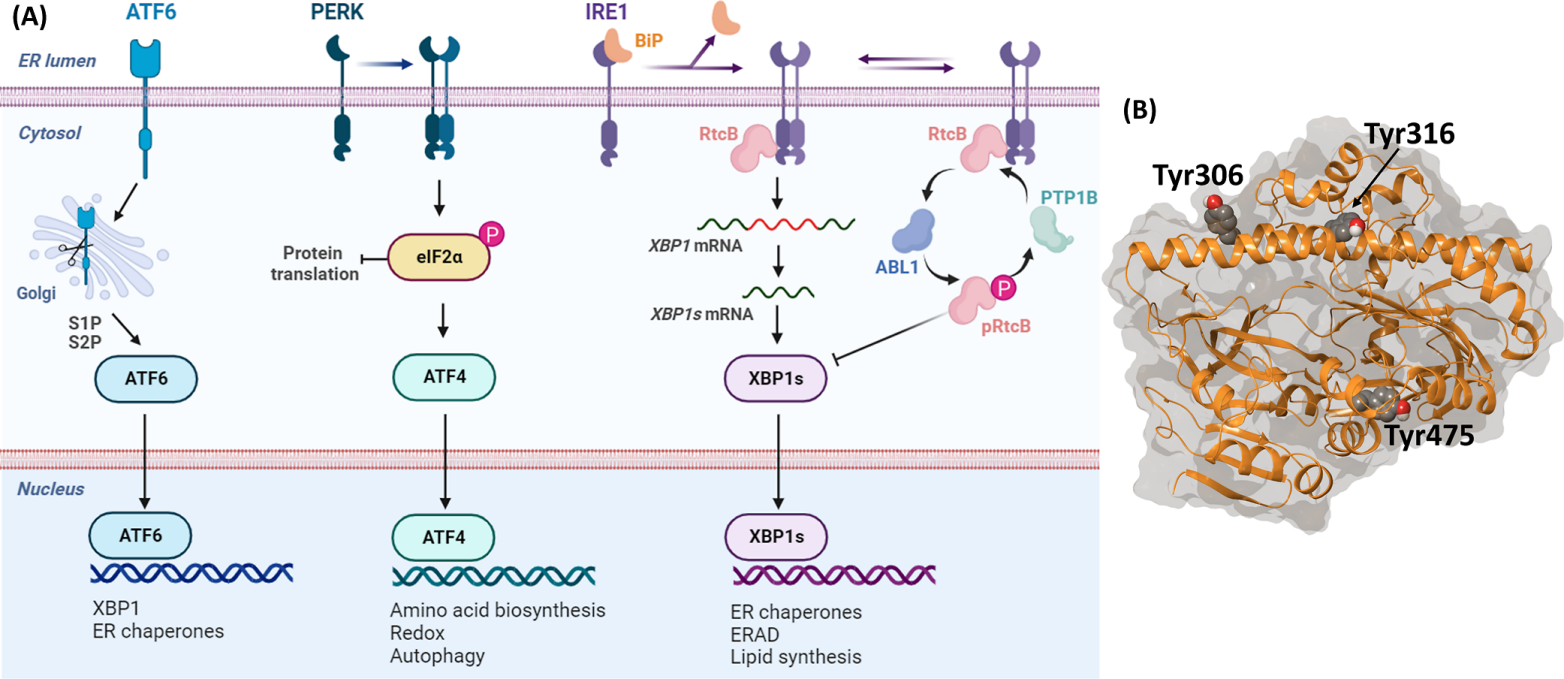
(A) The three transmembrane UPR sensors IRE1, PERK, and ATF6 are triggered by ER stress to restore cell homeostasis. The phosphorylation (by ABL1) and dephosphorylation (by PTP1B) cycles of the RtcB RNA ligase play a key role in the UPR outcome through the IRE1 branch. (B) The crystal structure of the RtcB ligase (PDB ID: 7P3B) and the location of three tyrosine residues (Y306, Y316, and Y475) which modulate the catalytic activity of the enzyme.

In the endoplasmic reticulum (ER),^1^ the unfolded protein response (UPR) is a cellular stress response predominantly controlled by three transmembrane sensors: inositol-requiring enzyme 1 (IRE1), protein kinase RNA-activated (PKR)-like ER kinase (PERK), and activating transcription factor 6 (ATF6)^2^ (Figure 1A). Once the protein folding capacity of ER is overwhelmed by cellular stress, the UPR is triggered by decreasing protein synthesis and increasing secretion and degradation of unfolded and misfolded proteins.^2^ IRE1 is the most evolutionarily conserved receptor of the three major sensors of UPR.^3,4^ Upon accumulation of unfolded or misfolded proteins in the ER,^5^ IRE1 undergoes oligomerization and autophosphorylation,^6^ with subsequent endoribonuclease activity.^2^ IRE1 catalyzes the splicing of a 26-nucleotide intron from human X-box-containing protein 1 (XBP1) mRNA to produce the spliced isoform XBP1s^7^ with the assistance of the tRNA ligase RtcB. XBP1s activates the transcription of a range of genes involved in ER-associated degradation,^8^ lipid biosynthesis, and protein folding to push the cell toward the survival state by restoring cell homeostasis.^2^ Therefore, the UPRosome and particularly the IRE1 signaling pathway have attracted much attention for the potential treatment of numerous diseases and disorders.

The RtcB ligase, also known as the RNA 2′,3′-cyclic phosphate and 5′-OH ligase, is a versatile enzyme involved in various cellular processes related to RNA metabolism, repair, and quality control. Its role in tRNA splicing and RNA repair is particularly crucial for maintaining the integrity and functionality of RNA molecules in the cell.^9^ RtcB is responsible for XBP1 mRNA splicing after IRE1-mediated cleavage.^10^ Recently,^11^ it has been shown that the catalytic activity of RtcB is modulated by the phosphorylation and dephosphorylation of three tyrosine residues (Y306, Y316, and Y475), respectively, conducted by the ABL1 tyrosine kinase and PTP1B phosphatase^12^ (Figure 1B). In particular, phosphorylation of RtcB at Y306 perturbs its interaction with IRE1, significantly attenuating XBP1 mRNA splicing.^11^ PTP1B is a well-studied phosphatase involved in many pathway control mechanisms, such as the downregulation of insulin,^13^ modulation of cell growth and proliferation,^14^ and regulation of leptin signaling.^15^ Moreover, an overexpression of this protein has been found in breast, prostate, and gastric cancers.^16^ The overexpression of PTP1B has been considered an effective prosurvival mechanism of cancer cells by activating the RtcB ligase.

**Figure 2:**
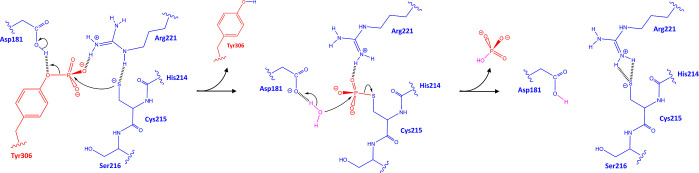
Two-step dephosphorylation reaction mechanism of PTP1B whereby a cysteine thiolate (Cys215) attacks the phosphate ester moiety of the substrate, resulting in a phosphocysteine intermediate, and a subsequent attack by water on the phosphocysteine intermediate leads to the release of the phosphate ion.

The catalytic mechanism of the PTP1B phosphatase has been elucidated with the help of the crystal structures and a phosphotyrosine-containing hexapeptide (DADEpYL) as a substrate, revealing a two-step reaction (Figure 2):^17^ (a) the PTP1B cysteine thiolate attacks the phosphate ester moiety of the substrate, resulting in a phosphocysteine intermediate, and (b) the subsequent attack of water on the phosphocysteine intermediate leads to the release of the phosphate ion. In a recent work^18^ using the fluorogenic 6,8-difluoro-4-methylumbelliferyl phosphate (DiFMUP) as a substrate, the second step of the reaction has been thoroughly studied using state-of-the-art computational approaches such as quantum mechanics molecular mechanics molecular dynamics (QM/MM MD) and metadynamics simulations, disclosing the important role of zinc and magnesium ions in the modulation of PTP1B through the second part of the reaction (hydrolysis of the phosphocysteine intermediate). However, neither the 3D structure and binding mode of the RtcB-PTP1B complex nor the underlying atomistic mechanism of the first part of the dephosphorylation reaction (phosphoryl transfer from the substrate to the enzyme) of RtcB has been elucidated so far. Therefore, in the current study, we addressed these missing pieces of the UPRosome machinery using advanced in silico methods such as our recently developed protein–protein docking meta-approach,^19^ quantum mechanics molecular mechanics well-tempered metadynamics (QM/MM WT-MetaD),^20^ and classical molecular dynamics (MD) simulations.

## 2 Methods

### 2.1 Protein Preparation

The crystal structures of the human PTP1B phosphatase (PDB ID: 2CM2, 1.5 Å)^21^ and RtcB ligase (PDB ID: 7P3B, 2.3 Å)^9^ were retrieved from the Protein Data Bank and prepared using the Schrödinger protein preparation wizard v2022-1.^22^ This includes the incorporation of hydrogen atoms and the modeling of possible missing side chains and loops using Prime.^23,24^ After fixing structural defects, water molecules were removed from the system, and the hydroxyl group on the side chain of the residue Tyr360@RtcB was replaced by a phosphate group. The hydrogen bonding network was optimized by adjusting the protonation and tautomeric states of relevant residues at pH = 7.4 using PROPKA [4]. Epik^25,26^ was used to assign the correct protonation states of the hetero entities at the same pH. Finally, the prepared structures were subjected to geometry refinement using the OPLS4 force field^27^ in a restrained structural minimization. The inactive conformation of the PTP1B phosphatase (with an open WPD-loop) was chosen to be able to study the early stage of PTP1B-RtcB complex formation, i.e., pTyr recognition, and phosphatase activation through the protein–protein docking calculation and restrained molecular dynamics simulation, respectively.

### 2.2 Protein–Protein Docking

**Figure 3:**
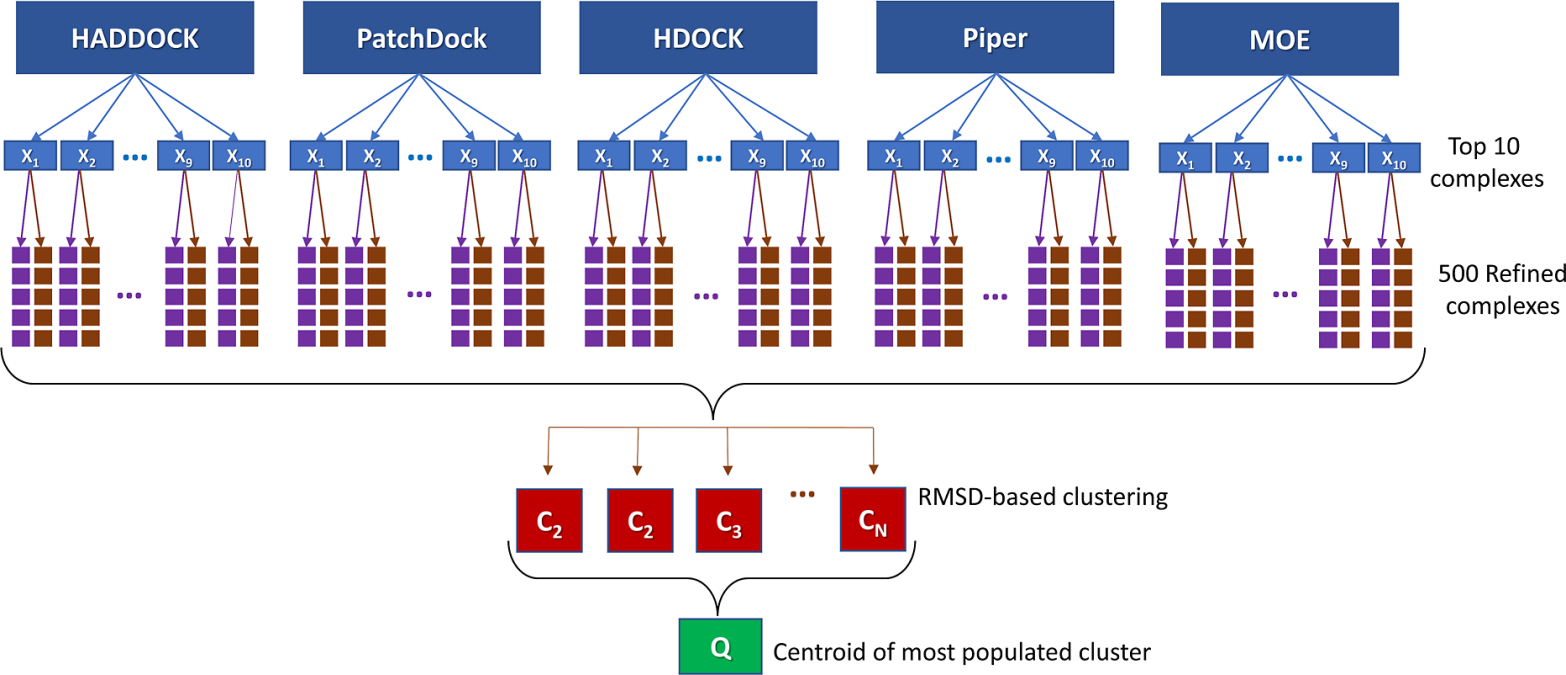
Flowchart of the protein–protein docking meta-approach used in this study to obtain a consensus protein complex from five different docking engines.

The protein–protein docking meta-approach was employed to integrate the benefits of five docking engines (HADDOCK,^28,29^ PatchDock,^30^ HDOCK,^31,32^ Piper,^33,34^ and MOE (Chemical Computing Group, Montreal)) to generate a “consensus-based” prediction of the RtcB-PTP1B complex. Table S1 provides a summary of the search algorithms and scoring functions used in each docking engine, along with their corresponding URL. The flowchart presented in Figure 3 shows the meta-approach used in this study.^19^ The docking protocol starts with performing a series of nonblind protein–protein docking calculations by introducing the interacting residues involved in the dephosphorylation reaction (i.e., pTyr306@RtcB and Cys215@PTP1B, Asp181@PTP1B, and Arg221@PTP1B). The default settings and parameters were used in all docking engines. The top ten predicted complexes (i.e., X_1_ to X_10_) from each docking engine were chosen, and each of the 50 docked poses underwent structural refinement using the GalaxyRefineComplex tool with two relaxation protocols.^35^ In the first protocol, only distance restraints were applied, while the second protocol applied both distance and position restraints. The five lowest-energy complexes from each refinement protocol were returned as the final 10 refined models for each initial complex. The total 500 refined complexes were clustered based on the root-mean-square deviation (RMSD) values of all heavy atoms using the Schrödinger program package (i.e., C_1_–C_N_) (Schrödinger v2022-1: Maestro, Schrödinger, LLC, New York, NY, 2020.). The optimum number of clusters, *N*, was determined from the Kelley penalty plots.^36^ Finally, the model nearest the centroid of the most populated cluster was considered the final model, *Q*.

### 2.3 MD Simulations

The consensus model from protein–protein docking was subjected to a 600 ns long restrained MD simulation in order to extend the conformational sampling and accelerate the activation of PTP1B where the WPD-loop alternates from the open to the closed conformation. In the restrained MD simulation, pTyr306@RtcB was guided toward the active site of PTP1B defined by the center of mass of the catalytic residue Cys215 and all residues within 3 Å of it (Leu110, Asn111, His214, Ser216, Gly218, Arg221, and Ser222). A harmonic attraction force of 0.01 kcal mol^–1^ Å^–2^ was applied to the backbone atoms of pTyr306 and the residues in the active site of PTP1B with a lower-wall cutoff distance of 5.0 ± 1.0 Å between the S atom of Cys215 and the P atom of pTyr306. The restrained MD simulation was followed by a 400 ns unrestrained MD run (1000 ns in total). The unrestrained MD simulation was clustered based on the backbone RMSD of the two proteins and used as the starting input structure in the subsequent QM/MM WT-MetaD simulations.

The MD simulations were conducted in the NPT ensemble using the Desmond engine^37^ with the OPLS4 force field^27^ and multitime-step RESPA integrator^38^ with bonded, near-nonbonded (short-range van der Waals forces), and far-nonbonded (long-range electrostatic forces) timesteps of 2.0, 2.0, and 6.0 fs, respectively. The RtcB-PTP1B complex was explicitly solvated in a cubic simulation box with a 10 Å buffer in each direction using the TIP3P water model^39^ and applying periodic boundary conditions in the MD simulations. The system was neutralized by incorporating an appropriate number of Na^+^/Cl^–^ ions, and a NaCl salt concentration of 0.15 M was considered. The Nosé–Hoover thermostat^40^ (relaxation time of 1 ps) and Martyna–Tobias–Klein barostat^41^ (relaxation time of 2 ps) were utilized to control the temperature and pressure of the system at 300 K and 1 atm, respectively. The long-range electrostatic forces were calculated using the particle-mesh Ewald method^42,43^ using default settings. The SHAKE algorithm^44^ was applied to the covalent bonds between all heavy atoms and hydrogens with maximum iterations and a tolerance of 8 and 10^–8^, respectively. The standard minimization and equilibration protocol was used as follows: (i) NVT Brownian dynamics with restraints on solute heavy atoms at *T* = 10 K for 100 ps, (ii) NVT simulations with a small time step at *T* = 10 K with restraints on solute heavy atoms for 12 ps, (iii) NPT MD simulations at *T* = 10 K with restraints on solute heavy atoms for 12 ps, (iv) NPT MD simulations at *T* = 300 K with restraints on solute heavy atoms for 12 ps, and (v) NPT MD simulations at *T* = 300 K without restraints for 24 ps.

It is worth highlighting that while protonation of Asp181 could be considered one step of the first part of the reaction, this residue was initially assigned in the protonated form at the outset of the MD simulation (open WPD-loop) due to the inherent limitations of modifying its protonation state during the course of the classical MD simulation. The protonation mechanism of Asp181@PTP1B during the conformational change from an open state to a closed state remains incompletely elucidated. Nevertheless, in the closed WPD-loop observed in the Michaelis complex (PDB-id: 1PTU),^17^ certain residues within the proximity of Asp181, specifically Lys120 and Glu115, in conjunction with water molecules, may collaborate to establish a hydrogen bonding network. This network is believed to play a pivotal role in facilitating the protonation of Asp181. However, additional research is necessary to substantiate this hypothesis, a task that falls beyond the scope of the current study.

### 2.4 QM/MM WT-MetaD

**Figure 4:**
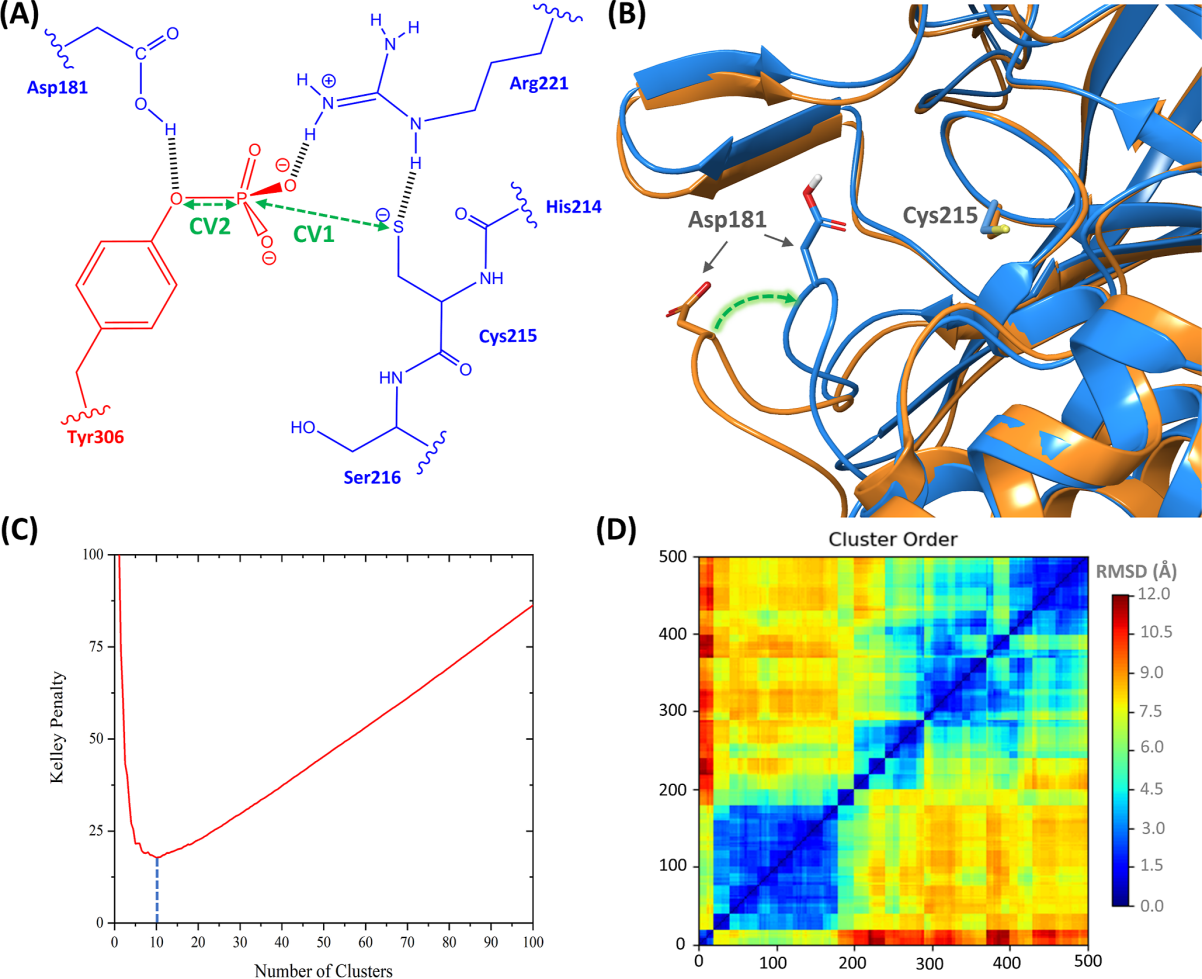
(A) QM subsystem along with the CVs used in the QMMM-WT-MetaD simulation. The blue and red residues belong to the PTP1B and RtcB protomers, respectively. (B) Superposed view of the inactive (orange colored ribbon, PDB ID: 2CMD) and active (blue colored ribbon, PDB ID: 1A5Y) PTP1B where the WPD-loop (highlighted by Asp181) alternates from open to closed conformation. (C,D) show the Kelley penalty plot and distance matrix heatmap of PTP1B-RtcB clustering, respectively.

The QM/MM WT-MetaD calculations were performed using the CP2K code^45,46^ version 9.1, patched with the enhanced sampling library PLUMED^47,48^ version 2.8. The QM subsystem (Figure 4A) includes the side chains of the residues pTyr306@RtcB, Asp181 and Arg221 of PTP1B, the entire Cys215@PTP1B, and part of His214 and Ser216 of PTP1B. The residue side chains were cut at the C_α_–C_β_ bonds such that the C_β_ and C_α_ atoms were part of the QM and MM regions, respectively, whereas the His214 and Ser216 residues were cut through the C–C_α_ bonds. The integrated molecular orbital molecular mechanics (IMOMM) method^49^ was employed to treat the linker atoms between the QM and MM subsystems. The rest of the system was considered part of the MM subsystem.

Exchange–correlation density functional theory (DFT) with the Perdew–Burke–Ernzerhof (PBE) generalized gradient approximation^50^ and DFT-D3(BJ) Grimme dispersion correction^51,52^ with a double-ζ valence plus polarization (DVZP) basis set^53^ and Goedecker, Teter, and Hutter (GTH) pseudopotentials^54^ was applied to the QM subsystem. This level of theory was successfully employed in studying various enzymatic reactions.^55−59^ It has been shown that the DFT-D3(BJ) Grimme dispersion correction provides better results for nonbonded interactions and more clear effects of intramolecular dispersion forces.^52^ The MM subsystem was treated by the side chain and backbone-modified AMBER14 force field (ff14SB)^60^ and the TIP3P water model.^39^ The missing force field parameters for Tyr306@RtcB were taken from the general AMBER force field (Y2P).^61^ Prior to the QM/MM-WT-MetaD simulation, the system was equilibrated by conducting 1 ns NVT followed by 1 ns NPT classical MD simulations with a fixed time step of 0.5 fs. The temperature and pressure were controlled using canonical sampling through a velocity rescaling (CSVR)^62^ at 298 K and a 1 atm thermostat and barostat, respectively. A cutoff distance of 10 Å was applied for nonbonded interactions, while the long-range electrostatic interactions were calculated using the smooth particle-mesh Ewald (SPME) method.^43^ A RMSD analysis was conducted to evaluate the potential for a significant conformational change by moving from OPLS4 (Desmond) to AMBER14 (CP2K). As Figure S1 illustrates, the RMSD analysis of the PTP1B-RtcB complex indicates a prompt convergence to an equilibrium state, characterized by minimal fluctuations oscillating around a notably modest mean value of 1.6 Å. This stability is further exemplified within the domain of interfacial residues, encompassing those residing in the proximity of the active site, where the average RMSD is impressively constrained at a mere 1 Å. Therefore, the move from OPLS4 to AMBER14 did not cause substantial conformational changes.

The collective variables (CVs) for the QM/MM WT-MetaD simulation were carefully chosen to fully represent the dephosphorylation reaction (Figure 4A): CV1 represents the nucleophilic attack of the negatively charged sulfur atom of Cys215 on the phosphorus atom of pTyr306, leading to the formation of the S–P bond. CV2 represents the breakage of the O–P bond and the release of Tyr306. To boost the sampling procedure of the CV space, the system was biased by adding a Gaussian kernel with an initial height of 0.5 kcal mol^–1^ and a width of 0.25 Å every 100 MD steps (50 fs). A biasing factor of 34.77 (i.e., 20 kcal mol^–1^) was set to scale the heights of the spawning Gaussian kernels. The rest of the settings were the same as those in classical MD. The QM/MM WT-MetaD simulations stopped when the convergence criteria were satisfied (90 ps in this case). Block analysis was used to monitor the convergence and calculate the errors in the free energy estimation. The diffusive behavior of CV sampling and the convergence in activation-free energy and reaction-free energy were considered convergence criteria.

### 2.5 Computational Resources and Performance

The QM/MM WT-MetaD simulation was carried out on a single node on the Dardel supercomputer, PDC, Stockholm. Each compute node consists of two AMD EPYC Zen2 7742 2.25 GHz 64-core processors along with 256 GB RAM, which implies that each compute node has a total of 128 physical CPU cores. Two virtual hardware threads are enabled on each physical CPU core, leading to a total of 256 logical/virtual cores. The performance of the QM/MM WT-MetaD simulation with 64 QM atoms and 170127 MM atoms is about 13 h ps^–1^.

## 3 Results and Discussion

The WPD-loop (residues 179–187) determines the catalytic activity of the PTP1B phosphatase as this loop alternates from the open (inactive) to the closed (active) conformation^17^ (Figure 4B). In particular, the residue Asp181 located in the WPD-loop plays a critical role as general acid and general base in the first and second parts of the reaction, respectively.^17,63^ Krishnan et al.^64^ showed that the Asp181Ala substrate-trapping mutant form of PTP1B lacks any catalytic activity. The structural and microenvironment analysis of Asp181 revealed a substantial change in the “solvent-accessible surface area (SASA)”, “desolvation massive”, and “effects local” parameters^65,66^ between the inactive (PDB ID: 2CMD) and active states (PDB ID: 1A5Y). The SASA parameter is a measure of the exposure of a residue to the solvent molecules or the degree of burial of that residue. The “desolvation massive” describes the energetic cost of removing water molecules from the immediate vicinity of a residue. A higher desolvation massive value indicates a higher energetic cost for desolvating the group, which is more energetically favorable when the residue is surrounded by fewer water molecules. The “effects local” term represents the contribution of the local chemical microenvironment to the p*K*_a_ value of a residue and is calculated as a relative energy difference between the protonated and deprotonated states in the presence of their neighboring chemical species. The “SASA”, “desolvation massive”, and “effects local” parameters of Asp181 were calculated to be 58.7 Å^2^, 0.0 kcal mol^–1^, and 0.21 and 13.4 Å^2^, 0.9 kcal mol^–1^, and 0.84 in the inactive (open WPD-loop) and active (closed WPD-loop) states, respectively. The “SASA” parameters indicate that Asp181 loses over 77% of its SASA after an inactive-to-active conformational change by being partially buried in the closed WPD-loop conformation. The “desolvation massive” values show that it is energetically favorable (0.9 kcal mol^–1^) for Asp181 to be surrounded with fewer water molecules in the closed conformation compared to the open conformation. The “effects local” terms indicate that the effect of the local chemical microenvironment on the protonation state of Asp181 in the active conformation is four times stronger than that of the inactive conformation. Overall, the p*K*_a_ value of Asp181 shifts significantly from 3.2 (deprotonated) in the inactive state to 7.5 (protonated) in the active state, which was elaborated by Bellomo et al.^18^ Due to the unique microenvironment surrounding the catalytic residue Cys215, it displays an unusually low experimental p*K*_a_ (∼4.6).^67,68^ Moreover, the “SASA” (4.5 Å^2^), “desolvation massive” (1.7 kcal mol^–1^), and “effects local” (0.28) parameters associated with the catalytic residue Cys215 in both open and closed conformations contribute to a diminished prediction of the p*K*_a_ value for this distinct residue.

### 3.1 Protein–Protein Docking and Initial Complex Formation

**Figure 5:**
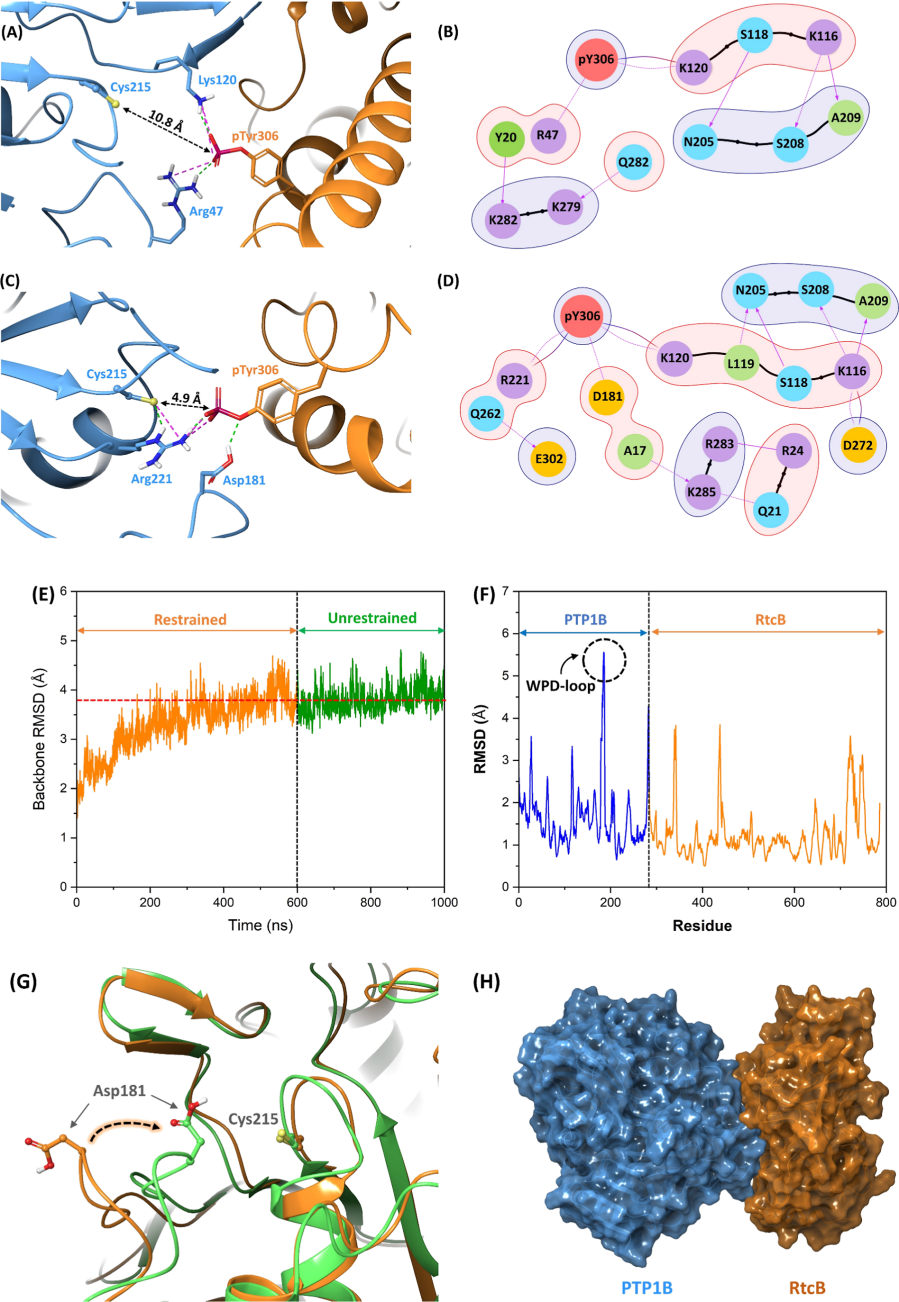
(A) Consensus RtcB-PTP1B complex model obtained from the protein–protein docking meta-approach and (B) interacting residues at the interface of the two proteins. (C) Representative RtcB-PTP1B complex after 400 ns unrestrained MD trajectory clustering and (D) interacting residues at the interface of two proteins in the representative complex. In (A) and (C), PTP1B and RtcB are shown in blue and orange ribbons, respectively. The S_Cys_–P_pTyr_ distance in the two complexes is indicated. (E) Backbone RMSD and (F) RMSF of the residues during the 1000 ns MD trajectory. (G) Closeup view of the first snapshot of the MD simulation (*t* = 0, orange ribbon) superposed on the last snapshot (*t* = 1000 ns, green ribbon), showing that the WPD-loop undergoes a substantial conformational change from open to closed conformation, leading to the activation of PTP1B. (H) Shape complementary to PTP1B and RtcB in the representative complex shown with blue and orange surfaces, respectively.

Protein–protein docking calculations using the meta-approach (Methods section) resulted in 10 clusters, where the most populated one consisted of 95 members. Figure 4C,D represents the Kelley penalty analysis and the associated distance matrix, respectively. Figure 5A,B illustrates the RtcB-PTP1B complex model nearest to the centroid of the most populated cluster and the interacting residues at the interface of the two proteins, respectively. As these figures show, pTyr306 of the RtcB ligase forms a hydrogen bond and salt bridge interactions with residues Arg47 and Lys120 of the PTP1B phosphatase. Arg47 is located in the pTyr recognition loop (residues 47–49) of PTP1B and is considered an important residue in the early-stage complex formation. Lys120 is a well-conserved residue in the lysine-loop (residues 119–121) of PTP1B that forms key interactions with the substrate.^17^ These strong hydrogen bond and salt bridge interactions between pTyr306 and the gate-keeper residues (Arg47 and Lys120) on the two sides of the active site make the phosphate group of pTyr306 properly oriented toward the catalytic residue Cys215 with an S–P distance of 10.8 Å (Figure 5A). The free energy of binding between PTP1B and RtcB proteins was calculated by employing the molecular mechanics generalized Born surface area (MM-GBSA) technique,^69^ giving a value of −51.3 kcal mol^–1^.

### 3.2 MD Simulations/Activation of PTP1B

The 600 ns restrained MD simulation was conducted on the consensus model obtained from protein–protein docking to further extend the conformational sampling and to explore the activation of PTP1B through the conformation change of the WPD-loop. The restrained MD was subsequently followed by 400 ns unrestrained MD to evaluate the complex stability and further postanalysis. Figure 5E,F shows the backbone RMSD and root-mean-square fluctuation (RMSF) of the residues during the full 1000 ns MD trajectory, respectively. The RMSD graph shows that the protein complex experienced most of its conformational changes during the first 400 ns of the restrained MD simulation, while it fluctuated around an average value during the last 200 ns. The plateau shape of the RMSD plot was observed throughout the 400 ns unrestrained MD simulation, indicating that the protein complex has reached a stable structural equilibrium with an average RMSD of 3.8 Å compared to that of the initial structure (Figure 5E). The RMSF plot indicates that the most fluctuating residues are those located in the WPD-loop, including Asp181 (Figure 5F). Figure 5G illustrates a closeup view of the first snapshot of the MD simulation (*t* = 0, orange ribbon) superposed on the last snapshot (*t* = 1000 ns, green ribbon), showing that WPD-loop underwent a substantial conformational change from open to closed conformation, leading to the activation of PTP1B. There are three key noncovalent interactions (NCIs) stabilizing the closed WPD-loop conformation, as described by Cui et al.,^70^ including: (1) the type II reverse turn defined by the hydrogen bond formed between Pro180 and Gly183, (2) the CH-π interaction between Trp179 and Pro185, and (3) the N-capping hydrogen bond between Ser187 and Phe191. Figure S2A illustrates the WPD-loop at the end of the MD simulation, demonstrating that three key NCIs are formed. Figure S2B shows the native interaction profiles during the 1000 ns MD simulation for the three types of NCIs discussed above. As this figure indicates, the formation of NCIs commences around 400–450 ns and is well-maintained throughout the entire trajectory. Figure 5C,D shows the representative RtcB-PTP1B complex after clustering the 400 ns unrestrained MD trajectory and the interacting residues at the interface of the two proteins, respectively. As these figures show, in the closed WPD-loop, Asp181 forms a hydrogen bond with the phosphoryl oxygen atom of pTyr306 that is most likely the primary driving force for the conformational change of the WPD-loop. These observations align with the outcomes reported by Crean et al.,^63^ who conducted parallel tempering metadynamics simulations within the well-tempered ensemble (PT-MetaD-WTE). Arg221 forms strong hydrogen bond and salt bridge interactions with pTyr306 and the catalytic residue Cys215, keeping these residues in close proximity (4.9 Å), as observed experimentally.^17^Figure 5B,D clearly shows that there are larger numbers of interactions between RtcB and active PTP1B compared to those of the inactive one, leading to a higher free energy of binding (−98.5 and −51.3 kcal mol^–1^ for the active and inactive PTP1B, respectively).

### 3.3 QMMM-WT-MetaD Simulation

**Figure 6:**
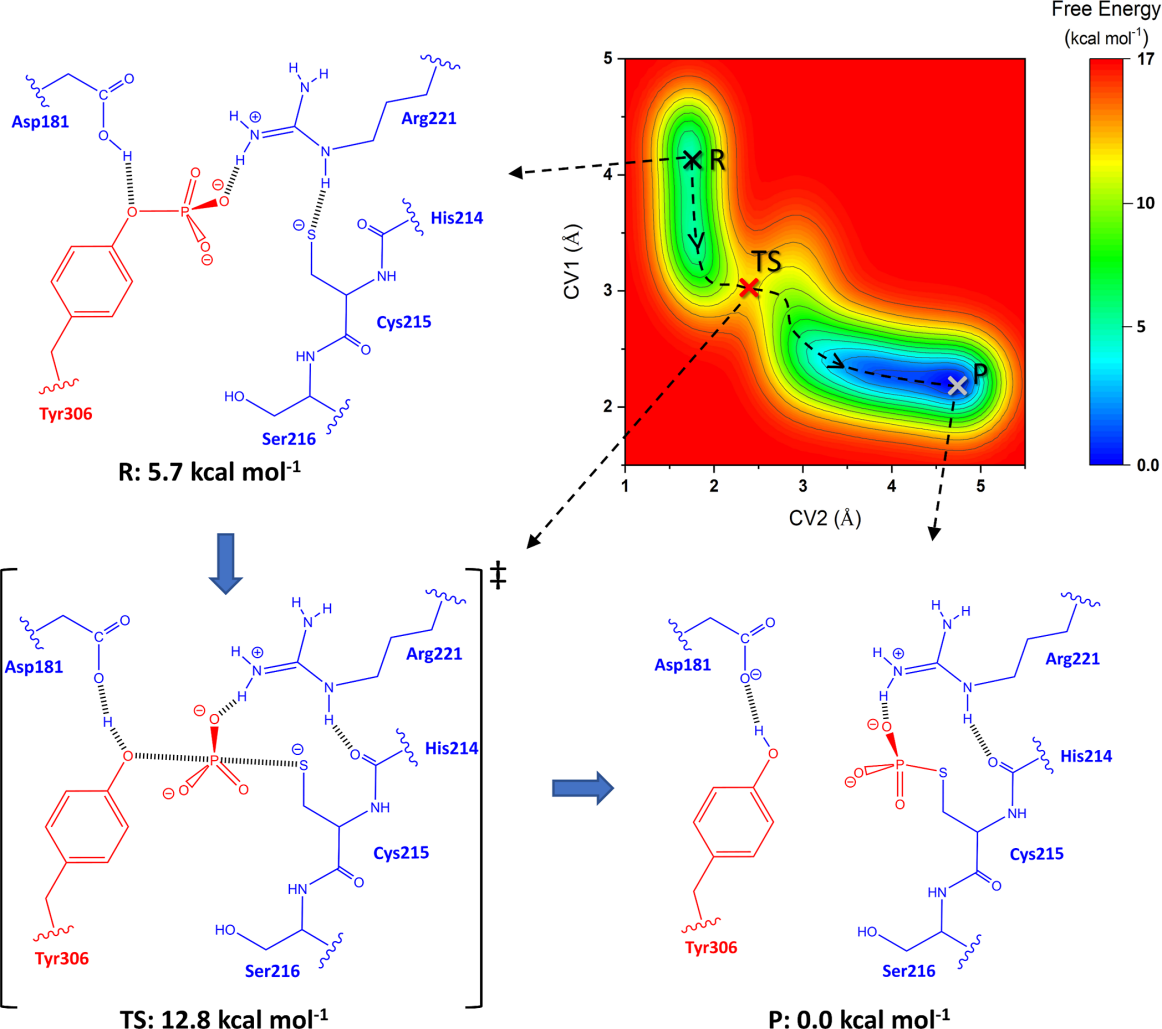
FES contour map of the first step of the reaction obtained from the 90 ps QMMM WT-MetaD simulation. The CV coordinates of the reactant, saddle point, and product are highlighted by black (point R), red (point TS), and gray (point P) crosses, respectively. The MEP is shown by a dashed line. The 2D structures of the reactant, saddle point, and product, along with their relative energies, are also presented.

Figure 6 shows the free energy surface (FES) contour map of the first reaction. The CV coordinates of the reactant, saddle point, and product are highlighted by black (point R), red (point TS), and gray (point P) crosses, respectively. The minimum free energy path (MEP) is shown by a dashed line. The 2D structures of the reactant, saddle point, and product along with their relative energies are also presented in the figure. As Figure 6 shows, the reactant state, point R, is located in a narrow but along CV1 elongated local minimum channel with CV1 = 3.38–4.28 and CV2 = 1.68 Å, and the associated relative free energy (Δ*G*_r_) is 5.7 kcal mol^–1^. The CV values are in good agreement with the O_S_–P (O_S_ refers to the oxygen atom of the serine residue in the Cys215Ser mutant) and O–P distances (3.3 and 1.6 Å, respectively) in the observed Michaelis complex in the crystal structure of the PTP1B Cys215Ser mutant with a DADEpYL hexapeptide substrate (PDB ID: 1PTU). DFT geometry optimization of pTyr using the M062X functional,^71^ the 6-311+G(d,p) basis set,^72^ and the integral equation formalism polarizable continuum model (IEFPCM) implicit solvent^73^ resulted in an equilibrium O–P distance of 1.73 Å (figure S3A). The DFT calculation was conducted using Gaussian 16 B.01.^74^ As Movie_1 in the Supporting Information shows, the hydrogen bond between Asp181 and pTyr306 frequently alternates between different oxygen atoms of the phosphate group. Arg221 maintains the hydrogen bond and salt bridge interactions with pTyr306 on one side and the catalytic residue Cys215 on the other, keeping the reactive residues in the proximity of the range of CV1 (3.38–4.28 Å).

Moving along the MEP, the transition state is located on a sharp saddle point with CV1 = 3.05, CV2 = 2.40, and Δ*G*_t_ = 12.8 kcal mol^–1^, leading to an activation free energy of Δ*G*^‡^ = Δ*G*_t_ – Δ*G*_r_ = 7.1 kcal mol^–1^. However, the CV values of the transition state are considerably different than the atomic distances observed in the transition state analogue (PDB ID: 3I7Z), where a tyrosine-modified vanadate complex was used to mimic the geometry of the phosphoryl transfer transition state. The corresponding values in the transition state analogue are 2.5 and 2.1 Å, respectively, clearly showing that the tyrosine-modified vanadate complex could not capture the true geometry of the transition state. The vanadate ion in the complex does not have the same charge, electronegativity, and atomic radius as the phosphate group in a substrate, which can affect the binding and orientation of the phosphoryl group and, in turn, lead to differences in the geometry of the transition state compared to that of the true substrate. The proton transfer from Asp181 to Tyr306 is initiated simultaneously as the phosphoryl group starts to leave. As Figure 6 shows, the proton atom lies at a distance midway in-between O_Asp181_–O_Tys306_ (1.18 and 1.24 Å for O_Asp181_–H and H–O_Tys306_ distances, respectively). Arg221 plays a crucial role in stabilizing the leaving group in the transition state. Cui et al.^70^ investigated the catalytic mechanism of PTP1B through a combined solution NMR and presteady-state kinetics experiments on wild-type and five WPD-loop mutants at 3.5 °C and a pH of 5.4 (as the reaction is too fast to be detected at room temperatures) using *p*-nitrophenyl phosphate (*p*-NPP) as a substrate. Their kinetic study resulted in a rate constant of 270 s^–1^ and an activation free energy of 13.1 kcal mol^–1^ derived from the experimentally observed rates using the Eyring equation. It has been shown, both experimentally^70^ and theoretically,^63^ that the catalytic rate in the PTP enzyme family is strongly correlated to the dynamics of the WPD-loop and one can therefore expect that the chemistry and loop motion are fully coupled. In such cases, a direct comparison to the experimental observation is thus not straightforward. Nonetheless, it must be acknowledged that the observed difference between the experimental and calculated activation free energy may also be attributed to the construction of the model adduct.

The product state is in a global minimum elongated along CV2 (Δ*G*_p_ = 0.0) with CV1 = 2.20 Å and CV2 = 3.51–4.68 Å. In this state, the proton is completely transferred from Asp181 to Tyr306 while the hydrogen bond interaction is maintained. The residue Arg221 keeps its hydrogen bond and salt bridge interactions with the phosphate group of the phosphorylated Cys215 (pCys215). The P–S bond length (CV2) in pCys215 is slightly longer than what is observed in the crystal structure of the PTP1B phosphoenzyme (1.92 Å; PDB ID: 1A5Y) and that calculated using the empirical valence bond method (1.98 Å).^75^ However, the DFT geometry optimization of pCys, with the same settings as those used for the pTyr optimization, resulted in an equilibrium P–S distance of 2.23 Å (Figure S3B). A P–S bond distance of 2.14 Å was observed for the phosphorylated cysteine residue pCys10 in a phosphotransferase enzyme using solution NMR (PDB ID: 1H9C).^76^

**Figure 7:**
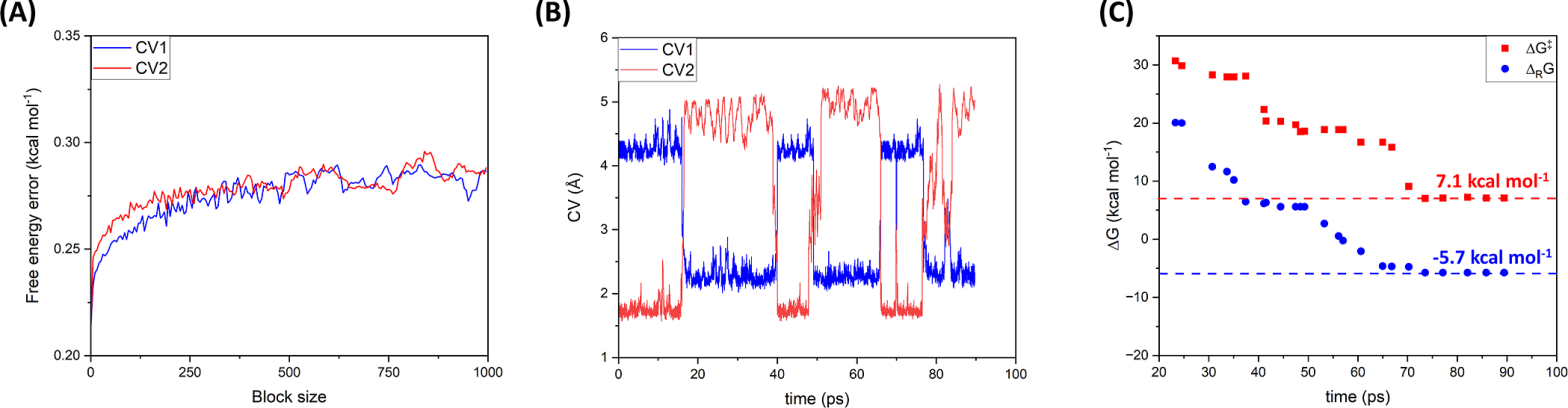
(A) Error in free energy estimation for CV1 and CV2 calculated by the block-average analysis explained in the main text. (B) Diffusive behavior of CV1 and CV2 sampling during the QMMM WT-MetaD simulation trajectory. (C) Convergence in activation free energy (Δ*G*^‡^) and reaction free energy (Δ_R_G) during the simulation.

Block-average analysis^77,78^ was used to analyze the convergence of the QMMM-WT-MetaD simulation and the associated statistical errors. In the block-average analysis method, the simulation trajectory is divided into a set of blocks with equal length. The error in the free energy value is estimated by comparing the average free energy values from each block. For a large number of blocks, the average error should be time-independent. Figure 7A shows the block-average analysis for CV1 and CV2 with 1000 blocks. As Figure 7A shows, the calculated errors in free energy for both CVs are perfectly converged to an average value of less than 0.27 ± 0.02 kcal mol^–1^. The block analysis (Figure 7A), along with the diffusive behavior of the CV sampling (Figure 7B) and the convergence in the activation free energy (Δ*G*^‡^) and reaction free energy (Δ_R_*G*) after ∼70 ps (Figure 7C), clearly confirms that the QM/MM WT-MetaD simulation has converged, at least within the vicinity of the initial enzyme conformation. However, there are some longer-time-scale motions of the enzymes that may affect the free energy profile beyond the 90 ps time scale.

The proton transfer from Asp181 to Tyr306 was not explicitly integrated into the QMMM-WT-MetaD simulation as a separate set of collective variables. The reason is that the negatively charged phosphate group and its electrostatic potential distort the regular donor–acceptor proton transfer in such systems. This impact would be pronounced within the buried zones where the solvent molecules have a limited ability to regulate and mask the electrostatic potential of the phosphate group. The most important impact of the phosphate group herein is a widening and flattening of the free energy profile of the proton transfer reaction. In the current system, the proton is nonlocalized and can move semifreely within the wide potential well generated mainly by the carboxylic oxygen atoms of Asp181 and the phosphate group of pTyr306. On the other hand, the movement of the proton within such a wide and flat potential well ensures a smooth forward–backward proton transfer without bias. A support for this claim is the fact that the CVs have been efficiently sampled during a 90 ps simulation featuring three barrier-crossing events (Figure 7B) with a low error in free energy estimation (Figure 7A).

To further validate this hypothesis, a reweighting calculation was conducted on the converged metadynamics trajectory using a new set of CVs. The first collective variable (CV1) is an antisymmetric combination of distances:
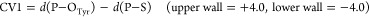
where *d*(P–O_Tyr_) and *d*(P–S) are the P–O_Tyr_ and P–S atomic distances, respectively (Figure S4a). The second collective variable (CV2) is a combination of coordination numbers, defined as
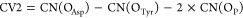
where CN(O_Asp_), CN(O_Tyr_), and CN(O_P_) are the coordination number of the carboxylic oxygen atoms of residue Asp181 (O_Asp1_ and O_Asp2_) with respect to H_Asp_, the coordination number of the atom O_Tyr_ with respect to H_Asp_, and the coordination number of the terminal oxygens of the phosphate moiety (O_P1_, O_P2_, O_P3_) with respect to H_Asp_, respectively (Figure S4a). An exponential decay switching function,
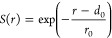
was used to measure the coordination numbers, where *d*_0_ and *r*_0_ have been set to 1.0 and 0.5 Å, respectively. Using this set of collective variables, we were able to fully deconvolute the proton transfer, although it was largely perturbed by the phosphate group. The free energy profile is shown in Figure S4b.

The activation energy associated with the minimum energy path connecting the reactant state (R) to the product state (P) passing through a transition state (TS) with CV1 = 0.65 and CV2 = 0.25 is estimated to be Δ*G*^‡^ = 7.9 kcal mol^–1^, which is only 0.8 kcal mol^–1^ larger than the activation energy estimated previously (7.1 kcal mol^–1^). There is a small activation energy (2.9 kcal mol^–1^) for the proton transfer from Asp181 to the terminal oxygen atoms of the phosphate moiety (O_P1_, O_P2_, and O_P3_) and reversibly. The protonated phosphate group can be identified by a wide/flat free energy profile in the ranges of CV1 = −3 to −1 and CV2 = −2 to −1 (highlighted by a circle in Figure S4b).

## 4 Conclusions

In this study, we have investigated the mechanism of RtcB ligase activation mediated by the PTP1B protein tyrosine phosphatase using advanced in silico methods. More specifically, we employed a protein–protein docking consensus approach, QMMM WT-MetaD, and classical MD simulations. Protein–protein docking analyses provide insights into the interaction between PTP1B and RtcB. Notably, phosphorylated Tyr306 forms hydrogen bonds and salt bridge interactions with the “gatekeeper” residues Arg47 and Lys120 of PTP1B. This finding confirms the significance of Arg47, located in the pTyr recognition loop (residues 47–49), in the early-stage formation of the complex. Subsequently, using classical MD simulations, we investigated the activation of PTP1B through the conformational change of the WPD-loop. The classical MD simulation revealed the critical role of Asp181 in driving the open-to-closed conformational change of the WPD-loop. To delve into the catalytic mechanism of dephosphorylation of RtcB by PTP1B, we employed QMMM-WT-MetaD simulations. The results demonstrated that the simulation was fully converged after 90 ps, as confirmed by block analysis, diffusive behavior of CV sampling, and convergence in activation free energy (Δ*G*^‡^ = 7.1 kcal mol^–1^) and reaction free energy (Δ_R_*G* = −5.7 kcal mol^–1^). Since DFT-based QM/MM-MetaD simulation is very demanding, the limited number of atoms in the QM region (64 atoms here) could be considered a potential limitation in our methodology. One might opt to employ the semiempirical-based QM/MM-MetaD simulation methodology as an effective means to accommodate a larger number of QM atoms and expedite computational throughput.^20^ However, the utilization of either DFT-based or semiempirical-based computations represents a substantial trade-off, necessitating a rigorous and deliberate assessment between precision and computational efficiency. The reasonable assumption of a “single initial reactant state”, where the most representative form of the reactant comes from the atomistic models of protein–protein docking, clustering, and relaxation, might affect the minimum energy pathway.

## 5 Data Availability

The pdb structures of the RtcB-PTP1B complex after protein–protein docking and after 1000 ns MD simulation, the 1000 ns MD trajectory, and a video captured from the QM/MM WT-MetaD simulation trajectory showing the reaction mechanism are provided freely at zenodo.org with DOI: 10.5281/zenodo.8099723.

## Supporting Information Available

Protein–protein docking engines employed; backbone RMSD analysis of the PTP1B-RtcB complex and interfacial residues during equilibration; WPD-loop and its key interactions during the MD simulation; DFT-optimized pTyr and pCys residues; and free energy profile and the new set of collective variables using reweighting calculations (PDF)
