# Supplementary material for: Multiscale In Silico Study of the Mechanism of Activation of the RtcB Ligase by the PTP1B Phosphatase
Source: J Chem Inf Model. 2024 Jan 29;64(3):905–17. doi: 10.1021/acs.jcim.3c01600 (PMC10865347; doi:10.1021/acs.jcim.3c01600)
Supplement: Supplementary file 1 — ci3c01600_si_001.pdf [file ci3c01600_si_001.pdf]

Supporting Information

Multiscale *in silico* Study of the Mechanism of Activation of RtcB Ligase by the PTP1B Phosphatase

Sayyed Jalil Mahdizadeh[1], Michael Stier[1], Antonio Carlesso[2,3], Aurore Lamy[1,4] , Melissa Thomas[1], and Leif A. Eriksson[*,1]

[1]*Department of Chemistry and Molecular Biology, University of Gothenburg, 405 30 Göteborg, Sweden*
[2]*Department of Pharmacology, Sahlgrenska Academy, University of Gothenburg, Gothenburg, Sweden*
[3]*Università della Svizzera italiana (USI), Faculty of Biomedical Sciences, Euler Institute, Via G. Buffi 13, CH-6900 Lugano, Switzerland.*
[4]*Department of Bioinformatics and Chemical Communication, Research Institute in Semiochemistry and Applied Ethology, Quartier Salignan 84400 Ap, France.*
[*]Corresponding author: Leif A. Eriksson
Email: leif.eriksson@chem.gu.se

Table S1. Summary of the searching algorithms and scoring functions implemented in the PP docking engines employed in this study.

PP Docking engine	Rearching algorithm	Scoring function	URL	Reference
**HADDOCK**	Flexible Monte Carlo search	Van der Waals, electrostatics, binding site restriction, and buried surface area	https://wenmr.science.uu.nl/haddock2.4/	[1]
**PatchDock**	Rigid body (geometric search, "LZerD" algorithm)	Shape complementarity, desolvation energy	http://bioinfo3d.cs.tau.ac.il/PatchDock/php.php	[2]
**HDOCK**	Rigid body FFT(template-based and ab initio template-free docking)	Long range shape complementarity and statistical mechanics-based	http://hdock.phys.hust.edu.cn/	[3]
**Piper**	Rigid body FFT and genetic algorithm	Shape complementarity, electrostatics, desolvation energy	https://www.schrodinger.com/	[4]
**MOE**	Rigid body Shape Complementarity "Triangle Matcher" algorithm	Shape complementarity, electrostatics, desolvation energy	https://www.chemcomp.com/Products.htm	-

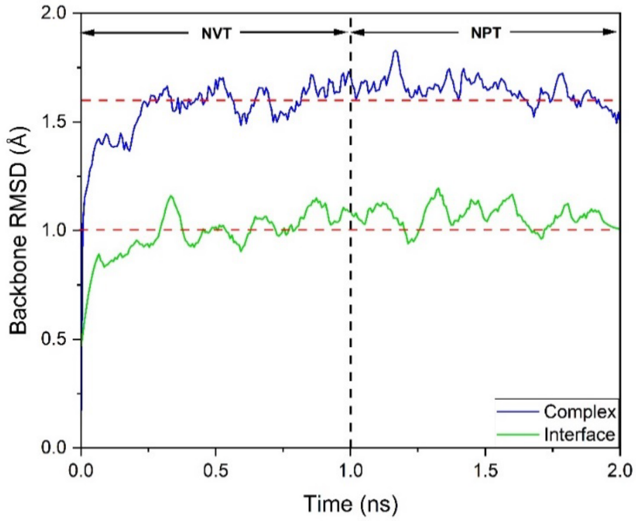

Figure S1: The backbone RMSD analysis of the PTP1B-RtcB complex (blue) and interfacial residues (green) during 1 ns NVT and 1 ns NPT equilibration prior to the production QM/MM WT-MetaD simulation.

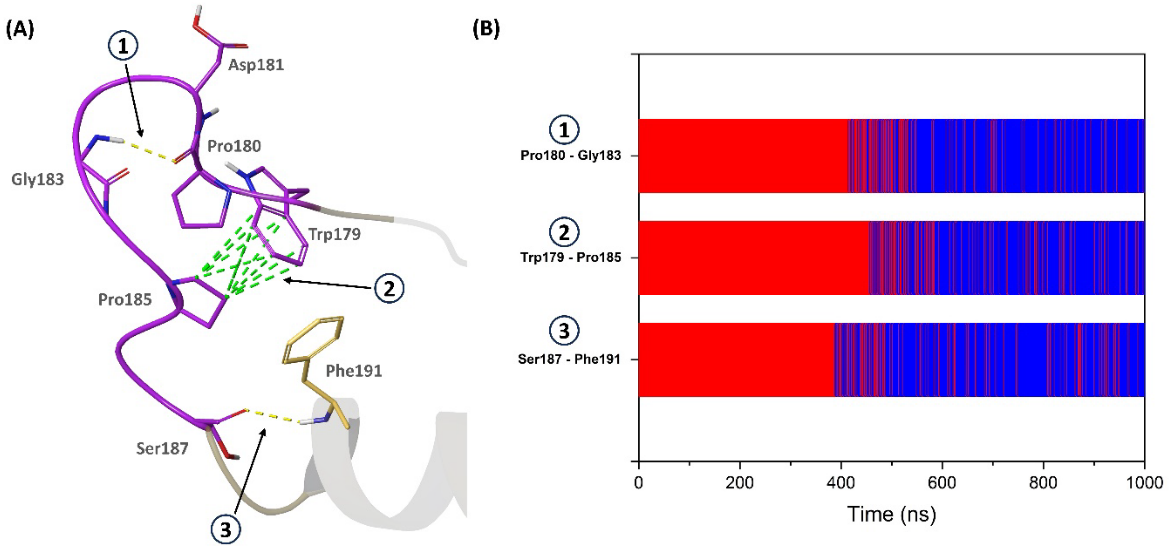

Figure S2: (A) WPD-loop at the end of the MD simulation demonstrating that three key NCIs are formed to stabilize the closed loop conformation [5]. (1) type II reverse turn defined by the hydrogen bond formed between Pro180 and Gly183, (2) CH-π interaction between Trp179 and Pro185, and (3) N-capping hydrogen bond between Ser187 and Phe191. (B) The native interaction profiles during 1000 ns MD simulation for three types of NCIs. The red and blue colors indicate that the specific interaction was diminished or maintained, respectively.

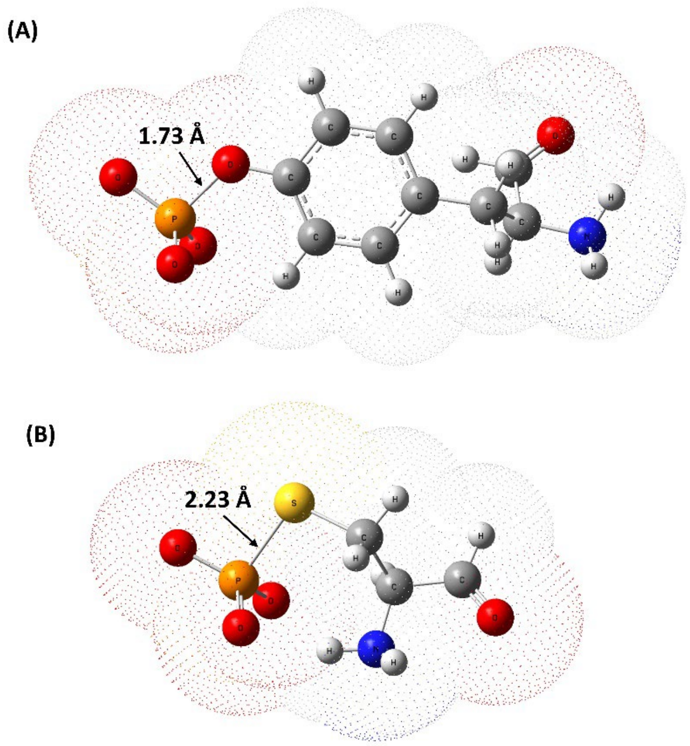

Figure S3. DFT geometry optimization of (A) pTyr and (B) pCys using the M062X/6-311+G(d,p) level of theory and IEFPCM implicit solvent model. The P–O and P–S bond distances are indicated. The IEFPCM solvation surfaces are shown as dots.

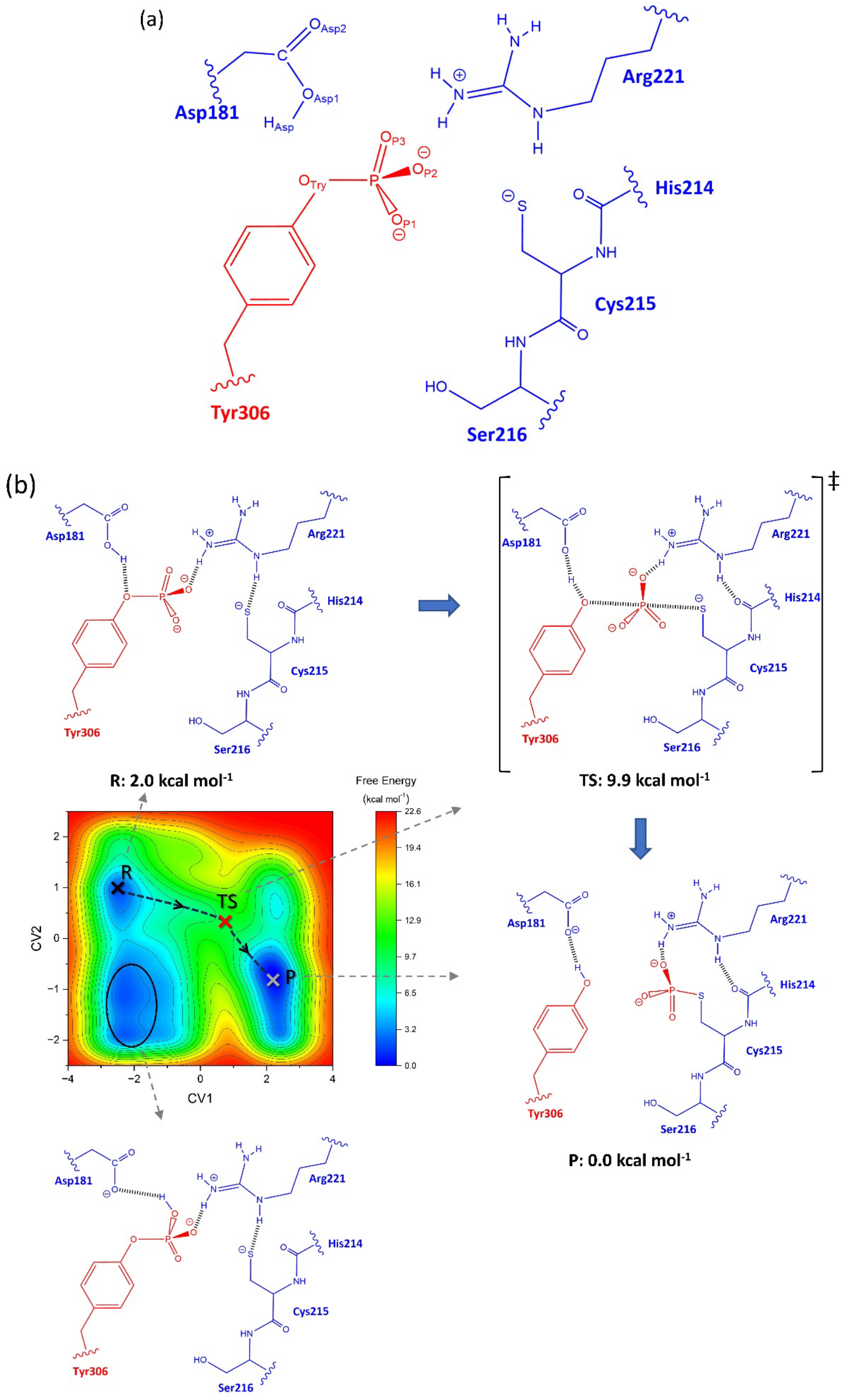
(a)
Asp181
Arg221
His214
Cys215
Tyr306
Ser216
(b)
R: 2.0 kcal mol-1
TS: 9.9 kcal mol-1
P: 0.0 kcal mol-1
Free Energy (kcal mol-1)
CV1
CV2

Figure S4. (a) Schematic representation of the atomic labels used to define the new set of collective variables. (b) Free energy profile elucidated using the new set of collective variables using reweighting calculations. The CV coordinates of the reactant, saddle point, and product are highlighted by black (point R), red (point TS), and gray (point P) crosses, respectively. The minimum free energy path (MEP) is shown by a dashed line. The 2D structures of the reactant, saddle point and product, along with their relative energies, are also presented.

References

[1] Dominguez, C.; Boelens, R.; Bonvin, A.M., HADDOCK: a protein− protein docking approach based on biochemical or biophysical information, *J. Am. Chem. Soc.* **2003**, 125, 1731-1737.
[2] Schneidman-Duhovny, D.; Inbar, Y.; Nussinov, R.; Wolfson, H.J., PatchDock and SymmDock: servers for rigid and symmetric docking, *Nucleic Acids Res.* **2005**, 33, W363-W367.
[3] Yan, Y.; Tao, H.; He, J.; Huang, S.-Y., The HDOCK server for integrated protein–protein docking, *Nature protocols*, **2020**, 15, 1829-1852.
[4] Kozakov, D.; Brenke, R.; Comeau, S.R.; Vajda, S., PIPER: an FFT-based protein docking program with pairwise potentials, *Proteins: Struct. Func. Bioinform.* **2006**, 65, 392-406.
[5] Cui, D.S.; Lipchock, J.M.; Brookner, D.; Loria, J.P., Uncovering the Molecular Interactions in the Catalytic Loop That Modulate the Conformational Dynamics in Protein Tyrosine Phosphatase 1B, *J. Am. Chem. Soc.* **2019**, 141, 12634-12647.
